# Preoperative Interventions for Alcohol and Other Recreational Substance Use: A Systematic Review and Meta-Analysis

**DOI:** 10.3389/fpsyg.2019.00034

**Published:** 2019-02-04

**Authors:** Luke Budworth, Andrew Prestwich, Rebecca Lawton, Alwyn Kotzé, Ian Kellar

**Affiliations:** ^1^School of Psychology, University of Leeds, Leeds, United Kingdom; ^2^Bradford Institute for Health Research, Bradford Royal Infirmary, Bradford, United Kingdom; ^3^Department of Anaesthesia, Leeds Teaching Hospitals NHS Trust, Leeds, United Kingdom

**Keywords:** alcohol consumption, illicit drugs, perioperative care, postoperative complications, preoperative period, substance-related disorder

## Abstract

**Background:** Preoperative alcohol and other recreational substance use (ORSU) may catalyze perioperative complications. Accordingly, interventions aiming to reduce preoperative substance use are warranted.

**Methods:** Studies investigating interventions to reduce alcohol and/or ORSU in elective surgery patients were identified from: Cochrane Library; MEDLINE; PSYCINFO; EMBASE; and CINAHL. In both narrative summaries of results and random effects meta-analyses, effects of interventions on perioperative alcohol/ORSU, complications, mortality and length of stay were assessed.

**Primary Results:** Nine studies (*n* = 903) were included. Seven used behavioral interventions only, two provided disulfiram in addition. Pooled analyses found small effects on alcohol use (*d*: 0.34; 0.05–0.64), though two trials using disulfiram (0.71; 0.36–1.07) were superior to two using behavioral interventions (0.45; −0.49–1.39). No significant pooled effects were found for perioperative complications, length of hospital stay or mortality in studies solely targeting alcohol/ORSU. Too few interventions targeting ORSU (*n* = 1) were located to form conclusions regarding their efficacy. Studies were generally at high risk-of-bias and heterogeneous.

**Conclusions:** Preoperative interventions were beneficial in reducing substance use in some instances, but more high-quality studies targeting alcohol/ORSU specifically are needed. The literature to date does not suggest that such interventions can reduce postoperative morbidity, length of hospital stay or mortality. Limitations in the literature are outlined and recommendations for future studies are suggested.

## Introduction

Up-to one in four adults in England drink hazardously, with up to 6% being alcohol dependent (Health Social Care Information Centre, 2015). Up-to 5% of people aged 16–59 (9.1% of 16–24 years old) use an illicit substance at least monthly (Home Office, 2015). The prevalence of these behaviors may be higher in elective surgical patients, particularly for alcohol: ~20% of elective surgical patients show features of alcohol dependency (Harris et al., 2008, 2011; Kip et al., 2008; Kleinwächter et al., 2010; Bradley et al., 2011) and 7.5%-20% of elective patients admit using other recreational substances at least once per year (up-to 26.4% of 18–30 years-old) (Kleinwächter et al., 2010; Kork et al., 2012).

Preoperative alcohol overuse is deleterious to surgical outcomes, with a 56% increase in postoperative general morbidity observed in a meta-analysis comparing risky alcohol users (defined as >36 grams per day of alcohol) vs. abstainers or non-risky users (studies including patients with “*clearly defined heavy drinking*” showed a 168% increased risk of mortality) (Eliasen et al., 2013). The specific adverse effects of excessive perioperative alcohol use include biochemical and hematological abnormalities (even in the absence of liver disease), altered drug kinetics and acute withdrawal (Chapman and Plaat, 2009). Preoperative use of other recreational substances (ORSU) has also been shown to be detrimental, though evidence is nascent: cannabis can potentiate or antagonize anesthetic drugs, presenting considerable risks (Dickerson, 1980; Mallat et al., 1996; Symons, 2002; White, 2002; Kuczkowski, 2004; Sharma et al., 2012); cocaine and amphetamine present cardiovascular issues (including intraoperative deaths) (Samuels et al., 1979; Steadman and Birnbach, 2003; Inouye et al., 2004; Skerman, 2005; Perruchoud and Chollet-Rivier, 2008; Baxter and Alexandrov, 2012; Elkassabany et al., 2013); and opioid users vs. non-users, independent of various comorbidities and surgical procedures, have up-to four times the odds of dying and two times the odds of suffering any morbidity in the perioperative period (Menendez et al., 2015). Lastly, a myriad of novel psychoactive substances have flooded the European market, many of which are demonstrably easy to acquire (e.g., legally) (Martinotti et al., 2015). The pharmacokinetic profile of many are unknown which may present potentially unpredictable risks and challenges for perioperative management.

For tobacco smoking it has been demonstrated that interventions to support patients in quitting are effective at modifying surgical outcomes (Theadom and Cropley, 2006; Thomsen et al., 2014). Intervention components, delivery, modes and contexts have also been studied, showing that intervention efficacy may be optimized by the use of specific behavior change techniques and other intervention characteristics (Prestwich et al., 2017). Reducing alcohol use from four, to two standard drinks a day may reduce the incidence of perioperative complications by up-to 50% (Tønnesen et al., 2009). Prolonged bleeding time and surgical stress responses may be reversed by an abstinence period of 4 weeks (Tønnesen et al., 2009). While there are no similar guidelines for ORSU, preoperative abstinence or reduction in use could arguably incur similar benefits. Both alcohol and ORSU may thus be modifiable risk factors for poor surgical outcomes. Consequently, interventions aimed at supporting surgical patients to modify their use are warranted, to protect patients and reduce healthcare costs (Scott et al., 2005).

Two previous reviews of preoperative interventions addressing alcohol use have shown that interventions may be effective at reducing drinking in surgical patients (Oppedal et al., 2012; Fernandez et al., 2015). However, the first had very strict inclusion criteria (e.g., including only randomized controlled trials), limiting the scope of the review, and undertook their literature search in 2011. The second included only behavioral interventions (excluding pharmaceutical support), undertook their literature search in 2013, and did not calculate pooled effects. To our knowledge, there have been no previous reviews assessing the efficacy of preoperative interventions for ORSU (e.g., cannabis, cocaine and/or opioid use). Additionally, psychological determinants (e.g., motivation to abstain) of alcohol and/or ORSU in the surgical context, and details of how users may be supported in behavior change, have not been described in sufficient detail in previous reviews.

In this review, we aimed to update previous alcohol intervention reviews and identify characteristics of effective (or ineffective) interventions. We also systematically review the literature on ORSU interventions, assessing quantitative evidence of their efficacy or otherwise at reducing substance use, and/or perioperative complications. Thirdly, we assess whether interventions could modify psychological determinants which may predict cessation; and lastly, try to characterize intervention components of any behavioral interventions using a behavior change taxonomy (Michie et al., 2013), so future researchers can more precisely replicate successful interventions.

## Methods

### Search Strategy

The Cochrane Library, PSYCINFO, EMBASE, MEDLINE, and CINAHL were searched up to November 2015. An additional scoping search was conducted in April 2017. Terms included “alcohol use,” and “preoperative care” (see Appendix 1). Additional studies were located via hand searching reference lists of included studies and relevant narrative reviews. Titles and abstracts were screened for eligibility by one reviewer, before a random sample (*n* = 200) were screened by two others side-by-side with open discussion (high level of agreement [*k* = 0.87]). Disagreements were resolved through open discussion. Full-texts of potential studies were inspected by two reviewers, with full agreement regarding their eligibility.

### Eligibility and Inclusion

Interventions targeting (i) alcohol and/or ORSU in the preoperative period (with or without postoperative maintenance sessions), and/or (ii) cognitive/behavioral determinants of alcohol and/or ORSU in (iii) elective patients only, were included. Any designs, surgical populations, and comparator groups (or lack thereof) were eligible.

### Primary Outcomes

1. Level (e.g., amount, frequency) and/or severity (e.g., dependence scores) of alcohol and/or ORSU at any time point (pre-surgery and/or post-surgery);
2. Perioperative: mortality (at any postoperative time-point), total complications (as defined by individual studies – at any postoperative time-point), and length of hospital stay (days);
3. Measures of psychological determinants of perioperative substance use (e.g., self-efficacy at reducing preoperative substance use).

### Secondary Outcomes

1. Other outcomes idiosyncratic to each study deemed clinically relevant, e.g., patient satisfaction, and quality of life.

### Risk-Of-Bias and Data Extraction

Risk-of-bias was assessed using the Cochrane Collaboration risk-of-bias assessment tool (Higgins et al., 2011). While it is acknowledged that other tools are often used to assess non-RCT designs, we felt it appropriate to appraise all studies against desired gold standard research methods. For all studies, risk-of-bias and data for primary and secondary outcomes, patient demographics, retention rates and study design features were single coded before being checked by a member of the review team. There was full agreement.

Behavior change techniques (BCTs) (Michie et al., 2013) targeted at alcohol or ORSU were coded by two authors to determine intervention components. Disagreements were resolved via discussion. Many intervention were poorly described. Studies with ambiguous descriptions of intervention components were coded “*social support (unspecified)*” (BCT 3.1) while BCTs for one intervention (Weinrieb et al., 2011) and all control groups were not coded due to insufficient reporting. BCTs outlined in this report, therefore, should be considered approximate.

### Statistical Analysis

Comprehensive Meta-Analysis (Borenstein et al., 2009) was used to compute, and where possible, pool effect sizes. Cohen's *d* was used as the effect size metric. For within-subjects outcomes, *d* > 0 favored post-intervention, *d* < 0 vice versa. For between-subjects outcomes, *d* > 0 favored intervention patients vs. controls, *d* < 0 vice versa.

Effect sizes were based on reported means and standard deviations, events in each group, or other statistical information, such as *t* or *p*-values. For some outcomes, it was not possible to calculate pre- to post-intervention between-subjects effect sizes. In these cases, post-intervention data were used. Unadjusted data were used where possible.

If outcomes were assessed at various time points, *d* for each was calculated—in addition to the average *d* across time points (pooling all in a within-study random effects meta-analysis). For studies using various measures for a related outcome, *d* for these measures were calculated individually—as well as the pooled *d* across them (pooled in a within-study random effects meta-analysis)—providing measure-specific *d*, and overall outcome-specific *d*.

Random effects meta-analyses were used to pool data for the primary outcomes (where possible). For each primary outcome with at least two studies, all studies were first meta-analyzed, before a series of sensitivity analyses investigated the results further—accounting for unique trial features. Only between-subjects data were used. The average *d* across within-study time points and measures assessing a related outcome were entered for each study (as detailed above). Homogeneity *Q* and *I*^2^ statistics assessed heterogeneity.

Though planned, the number of studies found was not sufficient to conduct metaregression assessing whether between-study intervention efficacy could be predicted from the use of different BCTs and certain design features.

## Results

### Characteristics of the Included Studies

**Figure 1:**
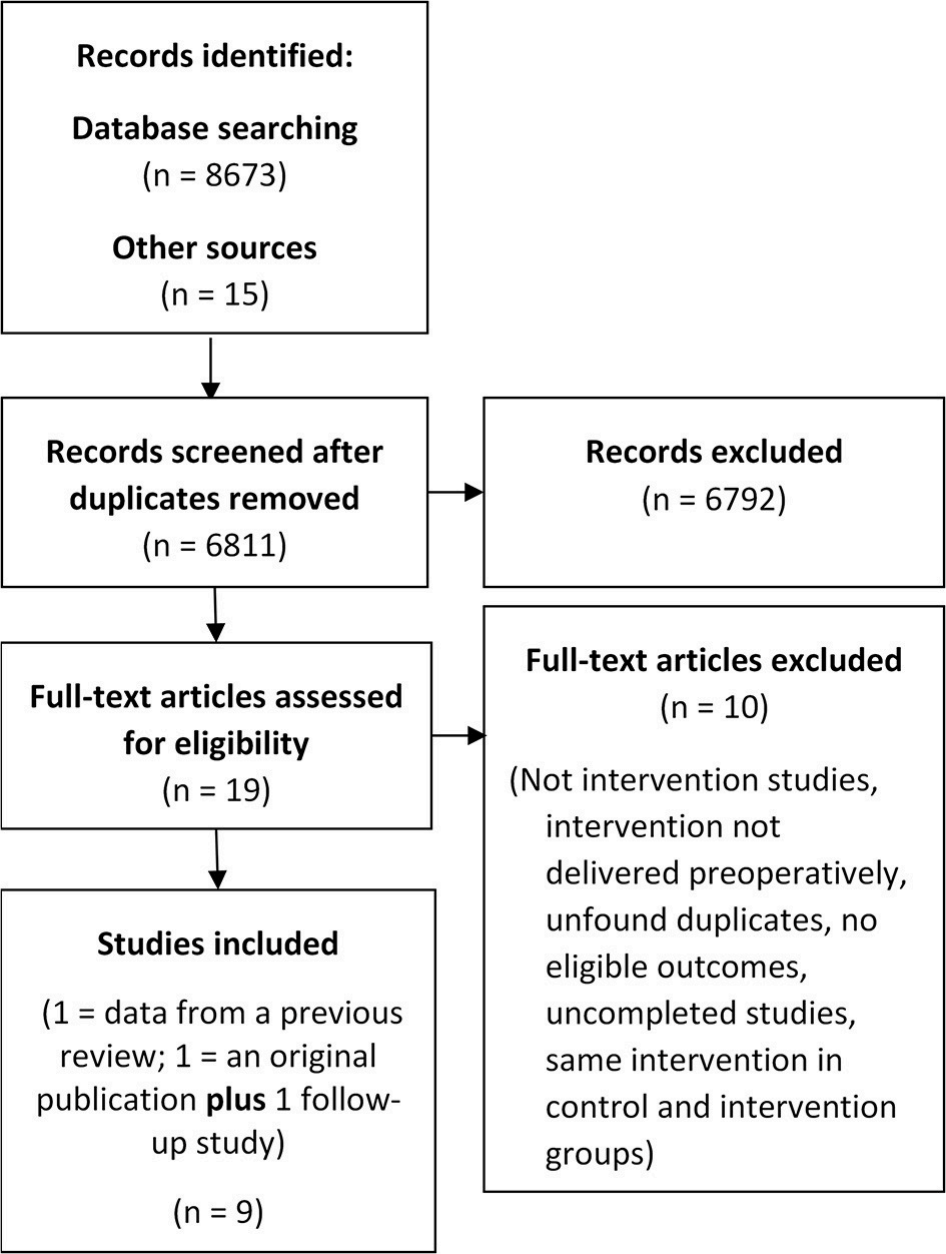
Flow diagram of the screening process.

From 8,688 studies identified, nine were eligible (Figure 1): five randomized controlled trials (Tønnesen et al., 1999; McHugh et al., 2001; Tønnesen, 2002; Kummel et al., 2008; Weinrieb et al., 2011) two non-randomized controlled trials (Shourie et al., 2006; Hansen et al., 2012) one pre- to post-intervention study (Ashton et al., 2013) and one mixed design study (Wyman et al., 2014) (which compared some outcomes between a control group [CG] and an intervention group [IG] and some within the IG only). Two studies targeted the use of alcohol and other recreational substances (Ashton et al., 2013; Wyman et al., 2014), the rest alcohol use only. Three studies aimed to modify other behaviors in conjunction with alcohol use e.g., exercise and smoking (McHugh et al., 2001; Kummel et al., 2008; Hansen et al., 2012). Five studies aimed to assess whether intervention could alter perioperative health outcomes (Tønnesen et al., 1999; Tønnesen, 2002; Shourie et al., 2006; Hansen et al., 2012; Wyman et al., 2014). See Table 1 for study characteristics, Table 2 for outcomes measures/results.

**Table 1:** Design, participant demographics, and intervention details.

**Publication information**	**Study design and sample size**	**Patient characteristics**	**Substance-related inclusion criteria**	**Patient substance use characteristics (baseline)**	**Intervention targets**	**Intervention description**	**Interventionist**	Intervention components (BCTs)^*^	**Intervention timing**
Ashton et al. (2013) (USA)	Pre- post-intervention study (“pilot study”) Total: 86	**Mean age** 46 **Gender** 32.6% male **Ethnicity** *White* 65.1% *Black* 29.5% *Other* 5.4% **Surgery** Bariatric	Patients with a history of substance misuse or at-risk substance use	22% positive for AUD (AUDIT-C). 43% abstinent from alcohol. 1 patient current cannabis user	**Determinants of substance use**	A brief group-based psychosocial intervention consisting of “*psychoeducation and discussion about the effects of substances and addictions after surgery*”	Psychologist, or post-doctoral psychology fellow	1.2; 3.1; 5.1; 11.2 (no specific protocol cited)	1 session 90 min long Time before surgery unknown
Hansen et al. (2012) (Denmark)	Controlled trial Total: 132 IG: 78 CG: 54	**Mean age** IG: 68 CG: 69 **Gender** IG: 43.6% male CG: 61% male **Ethnicity** Not reported **Surgery** Hip and knee arthroplasty	Males drinking >21 units, female drinking >14 units of alcohol per week	**IG:** 4 patients identified as risky drinkers **CG:** not stated	**Alcohol use** Healthy eating Exercise Smoking Medication use	**IG:** A brief one-to-one preoperative “*motivational conversation”* intervention with tailored information, recommendations and referral to specialists **CG:** Treatment as usual (no further elaboration)	Nurse	5.1; 9.1 (no specific protocol cited)	1 session Unknown duration 31 days before surgery
Kummel et al. (2008) (Finland)	Randomized controlled trial Total: 117 IG: 49 CG: 68	**Mean age** IG: 72 CG: 72 **Gender** IG: 69% male CG: 75% male **Ethnicity** Not reported **Surgery** Coronary artery bypass	None	**IG:** 40% alcohol use > once weekly, 47% 1–2 times a month **CG:** 44% > once weekly, 31% 1–2 times a month	**Alcohol use** Self-care Exercise Healthy eating	**IG:** A multi-session group counseling and guidance intervention, with “*adjustment education*” **CG:** Treatment as usual (no further elaboration)	Nurse	3.1 (no specific protocol cited)	5 sessions, 1 before surgery, 4 after Time before surgery unknown Duration unknown Unclear when or how many times alcohol use addressed
McHugh et al. (2001)^**^ (UK)	Randomized controlled trial Total: 98 IG: 49 CG: 49	**Median age** IG: 61 CG: 63 **Gender** IG: 71.4% male CG: 79.6% male **Ethnicity** Not reported **Surgery** Coronary artery bypass	None	**IG:** mean (SD) = 6.9 (10.5) drinks weekly (8 g pure alcohol) **CG:** 6.6 (8.5) drinks weekly	**Alcohol use** Exercise Healthy eating	**IG:** A multi-session one-to-one health education intervention, with general advice/information, motivational enhancement techniques, and telephone support **CG:** Treatment as usual (no further elaboration)	Nurse	1.1; 2.3; 3.1; 9.1; 9.2 (no specific protocol cited)	Monthly sessions upto the month of surgery (average 8 months) Flexible in length Unclear when or how many times alcohol use addressed
Shourie et al. (2006) (Australia)	Controlled trial Total: 136 IG: 91 CG: 45	**Mean age** IG: 55 CG: 52 **Gender** IG: 91.1% male CG: 74.4% male **Ethnicity** Not reported **Surgery** Mixed	Males drinking >60 g, females drinking >40 g alcohol daily	**IG:** mean (SD) = 8.2 (1.9) AUDIT-C score, 66.8 (43.9) grams of alcohol per day. 13.3% alcohol dependent (DSM interview diagnosed) **CG:** 8.3 (1.7) AUDIT-C score, 73.7 (50.1) grams per day	**Alcohol use**	**IG:** A brief one-to-one motivational enhancement based intervention named the “*Drink Less*” programme Dependent users referred to alcohol specialists for withdrawal management **CG:** Treatment as usual (“*a description of process of admission, preparation for, and recovery from surgery and written material as routinely provided by each surgeon and/or hospital*”)	Member of research team	1.1; 1.2; 2.3; 3.1; 3.3; 5.3; 5.6; 8.1; 12.2; 12.3; 15.1 (protocol cited)	1 session Unknown duration At minimum, more than 7 days before surgery
Tønnesen et al. (1999) (Denmark)	Randomized controlled trial Total: 41 IG: 20 CG: 21	**Median age** IG: 58 CG: 61 **Gender** IG: 100% male CG: 84% male **Ethnicity** Not reported **Surgery** Colorectal surgery	Patients drinking >60 g alcohol daily	**IG:** median (range) = 84 (60–480) grams of alcohol per day **CG:** 72 (60–480) grams per day	**Alcohol use**	**IG:** 800 mg Disulfiram taken under supervision twice weekly **CG:** Treatment as usual (no further elaboration)	Member of research team	11.1	Weekly up to the week before surgery
Tønnesen (2002) (Denmark)	Randomized controlled trial Total: 28 IG: 15 CG: 13	**Age range** Entire sample: 39–75 **Gender** IG: 100% male CG: 100% male **Ethnicity** Not reported **Surgery** Hip arthroplasty	Patients drinking >60 g alcohol daily, or 420 g weekly	**IG:** median (range) = 72 (60–156) grams of alcohol per day **CG:** 72 (60–96) grams per day	**Alcohol use**	**IG:** 400 mg Disulfiram taken under supervision, and 400 mg taken without supervision per week Brief weekly motivational counseling sessions Use of B vitamins Telephone support **CG:** Treatment as usual (no further elaboration)	Member of research team	3.1; 11.1 (no protocol for counseling intervention cited)	Weekly up to the week before intervention
Weinrieb et al. (2011) (USA)	Randomized controlled trial Total: 91 IG: 46 CG: 45	**Median age** IG: 51 CG: 48 **Gender** IG: 85% male CG: 82% male **Ethnicity** *White* IG: 85%; CG: 78% *Other* IG: 15%; CG: 22% **Surgery** Liver transplant	Patients who had drank at least one alcoholic drink within 2 years preceding a liver transplant evaluation (who were previously alcohol dependent)	**IG:** median (range) = 20.5 (10–28) years of past alcohol misuse, 9 (6–15) months since last drink, 17.4% received illicit substance use treatment in the past **CG:** 15 (10–20) years of past misuse, 9 (418) months since last drink, 22% past illicit substance use treatment	**Alcohol use** (possibly **other recreational substance use**)	**IG:** A multi-session intervention consisting of “*motivational enhancement therapy*,” “*case management*” and “*encouragement to attend AA meetings*” **CG:** Treatment as usual consisting of referral to community AA and “*standard outpatient therapy*”	Member of research team	Uninterpretable (no specific protocol cited)	7 sessions 50 min long Time to surgery unknown
Wyman et al. (2014) (USA)	Controlled trial/pre-post (“pilot study”) Total: 174 IG: 107 CG: 67	**Mean age:** IG: 57 CG: 51 **Gender:** IG: 95.3% male CG: 91% male **Ethnicity:** *White* IG: 58.9%; CG: 73.1% *Black* IG: 35.5%; CG: 23.9% *Other* IG: 5.6%; CG: 3% **Surgery** Mixed	Patients scoring 6 or above on the AUDIT-C Patients who reported any other recreational substance use in the previous 6 months	**IG:** mean (SD) = 5.4 (3.4) AUDIT-C score, 39% used illicit substances in the previous 6 months, 43.4% previously treatment for substance use disorder **CG:** 4.7 (3.4) AUDIT-C score, 60.6% other recreational substance, 42.4% past recreational substance use treatment	**Alcohol use Other recreational substance use** (*per se*)	**IG:** A brief group-based psychosocial intervention based on motivational interviewing techniques, with referral to substance abuse treatment specialists if requested **CG:** Results of pre-assessment screening, and brief education on perioperative substance abuse, and advice to quit	Psychologist, social worker or nurse	1.1; 1.2; 1.3; 1.5; 2.2; 3.1; 9.1 (no specific protocol cited)	1 session 2 hours long 59 days before surgery on average

*AA, alcoholics anonymous; AUDIT-C, alcohol use disorder identification test—consumption; AUD, alcohol use disorder; BCT, behavior change technique(s); CG, control group; DSM, diagnostic and statistical manual of mental disorders; ENT, ear, nose and throat; IG, intervention group; SD, standard deviation; UK, United Kingdom; USA, United States of America; 1.1 Goal Setting (behavior); 1.2 Problem Solving; 1.3 Goal Setting (outcome); 1.5 Review behavior goal(s); 2.2 Feedback on behavior; 2.3 Self-monitoring of behavior; 3.1 Social support (unspecified); 3.3 Social support (emotional); 5.1 Information about health consequences; 5.3 Information about social and environmental consequences; 5.6 Information about emotional consequences; 8.1 Behavioral practice/rehearsal; 9.1 Credible source; 9.2 Pros and cons; 11.1 Pharmacological support; 11.2 Reduce negative emotions; 12.1 Restructuring the physical environment; 12.3 Avoidance/reducing exposure to cues for the behavior; 15.1 Verbal persuasion about capability*.

* *Behavior change techniques (BCTs) were reported for no study. Those noted here were coded post-hoc by the review authors where possible (not all could be coded due to poor reporting of intervention protocols)*.

** *In the secondary follow-up analysis of this study (Rideout et al., 2012) 110 patients were included*.

**Table 2:** Assessment timing, recruitment, retention, outcome measures, and results.

**Publication information**	**Assessment time points**	**Substance use and determinants**	**Perioperative complications**	**Secondary outcomes**	**Recruitment and retention**	**Summary of results: substance use and determinants**	**Summary of results: perioperative complications**	**Summary of results: secondary outcomes**
Ashton et al. (2013)	**Baseline:** Immediately pre-intervention (after being referred from pre-assessment) **Follow-up:** Immediately post-intervention	**Baseline and follow-up:** Intentions to use substances after surgery (“not likely”) Knowledge of the negative effects of postoperative substance use (quiz format, score/10) Coping strategies to aid postoperative substance use reduction (number noted)	–	**Follow-up:** Patient satisfaction (questionnaire)	**Recruitment:** Duration of recruitment not specified **Retention:** No dropouts	**Intentions:** *d: 0.97; 95% CI: 0.49–1.45* **Knowledge:** *d: 4.57; 95% CI: 3.85–5.28* **Coping:** *d: 2.05; 95% CI: 1.67–2.42*	–	**Patient satisfaction:** *High*
Hansen et al. (2012)	**Baseline** (at surgical pre-assessment) **Follow-up:** 3 months (via patient chart review)	–	**Follow-up:** Length of hospital stay Mortality Total complications “*Unintentional patients paths*”^*^ Readmissions Number of patients meeting discharge criteria late	**Baseline and follow-up:** Health related quality of life (EuroQuol 5d) Disease specific outcome score (DSOS)	**Recruitment:** Over 2 months for each group. 5.7% of eligible patients declined participation **Retention:** No dropouts	–	**Length of hospital stay:** *d: 0.51; 95% CI: 0.15–0.86* **Mortality:** *Zero deaths* **Total complications:** *d: 0.39; 95% CI: −0.19–0.97* **Unintentional patient paths:** *d: 0.37; 95% CI: −0.06–0.80* **Readmissions:** *d: 0.21; 95% CI: −1.33–1.75* **Late discharge:** *d: 0.72; 95% CI: −0.20–1.24*	**EuroQUOL 5d:** *d: 0.13; 95% CI: 0.22–0.48* **DSOS:** *d: 0.32; 95% CI: 0.03–0.67*
Kummel et al. (2008)	(via survey questionnaires sent to participants) **Baseline Follow-up:** 3, 6, and 12 months	**Baseline and follow-up:** Use of alcohol (none vs. ≥ once a week calculated)	–	**Baseline and follow-up:** Symptoms of angina pectoris (not at all vs. ≥ at least once a day) Functional abilities (questionnaire probing stair climbing ability)	**Recruitment:** Over 3.5 years. 38% approached met the inclusion criteria **Retention:** 68% completed all follow-ups	**Alcohol use (3 months):** *d: 0.09; 95% CI: −0.59–0.77* **Alcohol use (6 months):** *d: −0.22; 95% CI: −0.63–0.20* **Alcohol use (12 months):** *d: −0.18; 95% CI: −1.83–0.82*	–	**Angina symptoms:** *d: −0.05; 95% CI: −0.31–0.22* **Functional abilities:** *d: 0.29; 95% CI: 0.07–0.52* (pooled across time points)
McHugh et al. (2001) and Rideout et al. (2012)	(via visitation by liaison nurse at home, or at a general practice clinic) **Baseline Follow-up:** 15 months **Secondary follow-up**: 12 years post-randomization	**Baseline and follow-up:** Grams per week of alcohol	**Secondary follow-up:** Mortality (not strictly “perioperative”)	**Follow-up:** Patient satisfaction (questionnaire) **Baseline and follow-up:** Health status (e.g., pain, general health; SF-36) Depression and anxiety (HADS) Physical health (objective measures e.g., BMI, blood pressure etc.)	**Recruitment:** Over 15 months. 85% of patients approached participated **Retention:** 79% of the IG, 83% of CG retained in the primary study. All but 12 retained for long-term follow-up	**Alcohol use:** *d: 0.28; 95% CI: −0.12–0.68*	**Mortality:** *d: 0.32; 95% CI: −0.12–0.76*	**Patient satisfaction:** *High* **SF-36 (pooled across domains):** *d: 0.73; 95% CI: 0.55–0.90* **Depression and Anxiety:** *d: 0.73; 95% CI: 0.32–1.14* **Physical health:** *d* range = *0.34–0.85;* all *p < 0.05*
Shourie et al. (2006)	**Baseline** (at surgical pre-assessment) **Up to 5 days post-surgery** (via patient chart review) **Follow-up:** 6 months (via interview)	**Baseline and follow-up:** AUDIT-C score Grams per day of alcohol Number of DSM diagnosed alcohol-dependent patients (only follow-up data used to calculate effect size)	**Up to 5 days post-surgery:** Length of hospital stay Total complications Mortality **Follow-up:** Hospital admissions	**Follow-up:** Number of GP visits (past 6 months) Number of days off work sick (past 6 months)	**Recruitment:** Over 2 years, 10 months. 2,889 patients ineligible, 114 refused participation. 4.3% of those approached recruited **Retention:** 4 of the IG, 13 of the CG dropped out	**AUDIT-C:** *d: 0.27; 95% CI: −0.11–0.65* **Grams per day of alcohol:** *d: −0.03; 95% CI: −0.41–0.35* **Number of alcohol dependent patients:** *d: −0.11; 95% CI: −0.76–0.55*	**Length of hospital stay:** *d: 0.19; 95% CI −0.17–0.55* **Total complications:** *d: −0.48; 95% CI: −0.89–0.06* **Mortality:** *d: −0.40; 95% CI: −1.94–1.15* **Hospital readmissions:** *d: 0.27; 95% CI: −0.11–0.65*	**GP visits:** *d: 0.11; 95% CI: 0.27–0.49* **Days off work:** *d: 0.17; 95% CI: 0.21–0.55*
Tønnesen et al. (1999)	**Baseline Follow-up:** 1 month post-surgery	**Baseline and follow-up:** Number of non-hazardous drinkers Number of alcoholic drinks consumed a week	**Follow-up:** Length of hospital stay Mortality Total complications	–	**Recruitment:** Over 2.5 years **Retention:** 2 IG, 4 CG patients dropped out	**Number of non-hazardous drinkers:** *d: 0.38; 95% CI: −0.31–1.08***Drinks consumed:** *d: 0.88; 95% CI: 0.18–1.57*	**Length of hospital stay:** *d: 0; 95% CI: −0.61–0.61* **Mortality:** *d: 0.38; 95% CI: −0.99–1.75* **Total complications:** *d: 0.99; 95% CI: 0.24–1.74*	–
Tønnesen (2002)	**Baseline Follow-up:** 1 and 3 months post-surgery	**Baseline and 1 month post-surgery:** Number of non-hazardous drinkers Number of alcoholic drinks consumed a week **Baseline and 3 months post-surgery:** Number of alcoholic drinks consumed a week	**1 month post-surgery:** Length of hospital stay Mortality Total complications	–	**Recruitment:** Duration of recruitment unspecified. 1.5% of those approached were included **Retention:** 4 of the CG, 5 of the IG dropped out	**Number of non-hazardous drinkers:** *d: 1.61; 95% CI: −0.04–3.26* **Drinks consumed (1 month):** *d: 0.78; 95% CI: 0.01–1.55* **Drinks consumed (3 months):** *d: 0.65; 95% CI: −0.11–1.41*	**Length of hospital stay:** *d: 0; −0.74–0.74* **Mortality:** *d: 0.72; 95% CI: −1.09–2.54* **Total complications:** *d: 0.64; 95% CI: −0.23–1.51*	–
Weinrieb et al. (2011)	**Baseline Follow-up:** 12, 24, 48, 72, 96 weeks pre-surgery (maximum 108 weeks). Alcohol outcomes reported across all time points (up-to 108 weeks post-randomization, pre-surgery)	**All time points:** Number of drinks consumed Number of drinking days Number of drinks per drinking day Number of patients who drank before surgery Number of other recreational substance users (data between groups reported at follow-up only)	**All time points:** Stages of change scores (SOCRATES), domains of: “*ambivalence*,” “*recognition*,” and “*regarding taking steps”*	**All time points:** Health-related quality of life (MOS-SF 12) Depression and anxiety (BDI/BAI)	**Recruitment:** Duration of recruitment unspecified. All potential liver transplant candidates screened **Retention:** 66/91 dropped out up to the final follow-up (comparable drop out between groups at each time point)	**Total drinks consumed:** *d: 1.81; 95% CI: 0.64–2.98* **Number of drinking days:** *d: 1.73; 95% CI: 0.58–2.89* **Drinks per drinking day:** *d: 1.17; 95% CI: 0.10–2.24* **Number of patients who drank before surgery:** *d: −0.05; 95% CI: −0.57–0.47* **Number of other recreational substance users:** *d: −0.16; 95% CI: −1.02–0.7* SOCRATES^**^: IG had a significant decrease in *ambivalence* and *recognition* scores across time, vs. CG. *Regarding taking steps* did not change over time	–	MOS-SF 12^**^: *No significant differences* BDI/BAI^**^: *Very little between-group differences*
Wyman et al. (2014)	**Baseline** (at surgical pre-assessment) **Follow-up:** ~3 months post-surgery (82.75 days on average [range 14–308 days] (via telephone) **Post-discharge** (via patient chart review)	**Baseline:** Questionnaire items (i. illicit substance use in the past 6 months, ii. the past year and iii. non-prescribed pharmaceuticals use in the last 6 months) AUDIT-C **Follow-up (IG only):** Items: use of illicit substances/non-prescribed pharmaceuticals in the past 6 months AUDIT-C **Post-discharge:** Number of patients screened positive for illicit substances (day of surgery)	**Post-discharge:** Postponement/cancellation of surgery ICU admissions Total complications	**Follow-up:** Patient satisfaction (questionnaire)	**Recruitment:** Over 2.5 years. 56 of the CG, 105 of IG underwent surgery—thus were eligible for analysis **Retention:** Only IG followed up (18.1% dropped out)	**Other recreational substance use** *d: 1.43; 95% CI: 0.95–1.91***AUDIT-C:** *d: 1.14; 95% CI: 0.87–1.41***Positive for other recreational substances:** *d: 0.17; 95% CI: −0.21–0.55*	**Postponement/cancellation of surgery:** *d: 0.22; 95% CI: −0.08–0.52* **ICU admissions:** *d: −0.04; 95% CI: −1.37–1.30* **Total complications:** *d: 0.01; 95% CI: −0.41–0.42*	**Patient satisfaction:** *High*

*AUDIT-C, alcohol use disorder identification test (consumption); BAI, Beck's anxiety inventory; BDI, Beck's depression inventory; BMI, body mass index; CG, control group; DSM, diagnostic and statistical manual of mental disorders; DSOS, disease specific outcome score (functional ability questions relating to issues, such as walking distance and ability); EUROQUOL 5D (a measure of quality of life); GP, general practitioner; HADS, hospital anxiety and depression scale; ICU, intensive care unit; IG, intervention group; MOS-SF 12, medical outcome study short form (12 items; a measure of health-related quality of life); SF-36, short form health survey (a measure of health status); SOCRATES, stages of change readiness and treatment eagerness scale (a measure of motivation to receive alcohol treatment)*.

* Unintentional patient pathways defined as: “a path by which the patient did not reach the discharge criteria within 5 days (minor complications), or had any postoperative complication within 3 months (major complications) leading to a non-planned inpatient visit, was readmitted within 3 months irrespective of cause, or died within 3 months postoperatively irrespective of cause.”

** *Effect size could not be calculated*.

Across the studies, patients were awaiting various surgical procedures (e.g., liver transplant, colorectal and coronary bypass surgeries). Sample sizes ranged from 28 to 174 (total: 903), with a large gender bias (~72% male). Six studies did not report patients' ethnicity, of three that did (Weinrieb et al., 2011; Ashton et al., 2013; Wyman et al., 2014) the majority were white (69%). All patients were adults.

Five studies delivered multi-session interventions (Tønnesen et al., 1999; McHugh et al., 2001; Tønnesen, 2002; Kummel et al., 2008) (the rest one session only) and three (Kummel et al., 2008; Ashton et al., 2013; Wyman et al., 2014) involved group (as opposed to one-on-one) interventions. In three studies (McHugh et al., 2001; Kummel et al., 2008; Hansen et al., 2012) the intervention was delivered by nurses, in four (Tønnesen et al., 1999; Tønnesen, 2002; Shourie et al., 2006; Weinrieb et al., 2011) by a member of the research group, one (Wyman et al., 2014) with multiple interventionists (psychologist, social worker or nurse), and one (Ashton et al., 2013) a psychologist. All studies described a motivational or general psychosocial behavioral intervention of some sort, but none described their intervention in terms of a behavior change technique taxonomy (that is, the interventions may not have been standardized). In addition to “*motivational counseling*” Tønnesen (2002) prescribed 800 mg of disulfiram a week (400 mg taken under supervision, 400 mg unsupervised), B vitamins and offered chlordiazepoxide for withdrawal symptoms. Tønnesen et al. (1999) had patients receive 800 mg of disulfiram weekly (other support unspecified).

Six studies (Tønnesen et al., 1999; McHugh et al., 2001; Tønnesen, 2002; Shourie et al., 2006; Kummel et al., 2008; Hansen et al., 2012) reported that controls received treatment as usual or routine care. Weinrieb et al. (2011) reported that controls received treatment as usual plus referral to community Alcoholics Anonymous and “*standard intensive outpatient therapy*”; Wyman et al. (2014) that controls were informed that they were at high-risk due to their substance use and were advised to reduce or cease alcohol use. Ashton et al. (2013) did not have a CG.

### Risk-Of-Bias

**Figure 2:**
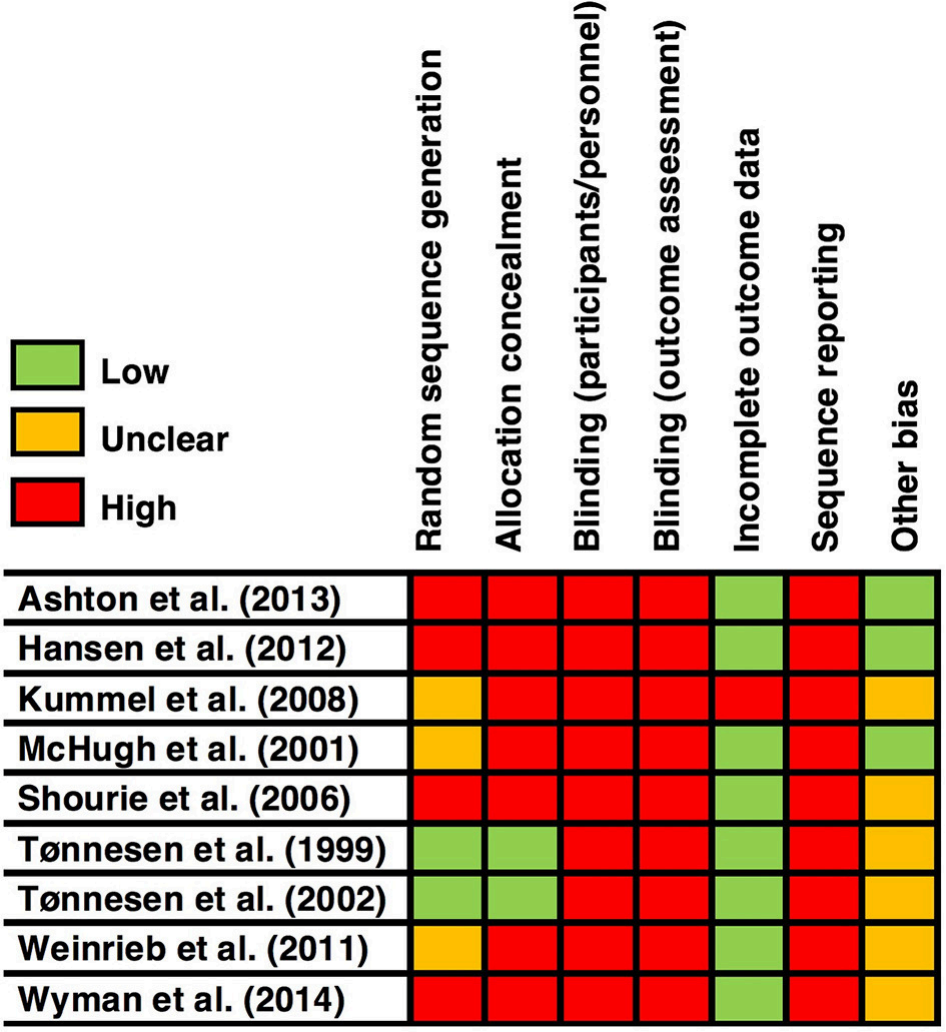
Risk-of-bias across individual studies.

Judgments can be seen in Figure 2.

#### Random Sequence Generation

Four studies (Shourie et al., 2006; Hansen et al., 2012; Ashton et al., 2013; Wyman et al., 2014) were deemed at high risk of bias as they did not use randomized designs. Three randomized studies (McHugh et al., 2001; Kummel et al., 2008; Weinrieb et al., 2011) were deemed at an unclear risk as they did not report how the randomization sequence was generated.

#### Allocation Concealment (Selection Bias)

All apart from two studies (Tønnesen et al., 1999; Tønnesen, 2002) data were deemed at high risk as they did not conceal allocations.

#### Blinding of Patients and Personnel (Performance Bias)

All studies were deemed at high risk of bias. Given the nature of the interventions (all requiring some degree of face-to-face contact), blinding was not achievable.

#### Blinding of Outcome Assessment (Detection Bias)

All did not report whether they (at minimum) blinded data analysts, all were deemed high risk.

#### Incomplete Outcome Data (Attrition Bias)

One study (Kummel et al., 2008) was deemed as high risk because no reasons for drop-outs were reported, the authors did not mention how many dropped out between groups, and when the authors compared drop-outs and non-drop-outs there were significant differences in certain variables (e.g., females' ages).

#### Selective Reporting (Reporting Bias)

All studies were stringently judged to be at high risk for selective reporting. We made this decision on the basis that no pre-registered protocol for any of the included studies could be found, thus there was a chance that authors could have consciously or unconsciously omitted any results inconsistent with their hypotheses. To locate pre-registered studies, each study article was checked for a passing reference or citation to a protocol, and the reviewers also noted any protocols found in the database and hand searches.

#### Other Bias

Six studies (Tønnesen et al., 1999; Tønnesen, 2002; Shourie et al., 2006; Kummel et al., 2008; Weinrieb et al., 2011; Wyman et al., 2014) were deemed at an unclear risk of bias due to a large bias in recruited patient demographics. The decision of unclear was made on the basis that it was unclear whether or how the particular variables could bias the results.

Five studies (Tønnesen et al., 1999; Tønnesen, 2002; Shourie et al., 2006; Weinrieb et al., 2011; Wyman et al., 2014) had a large gender bias toward males, and one study (Kummel et al., 2008) only recruited patients above 65 years-old.

## Results

Effect sizes across individual study outcomes are reported in Table 2.

### Alcohol Use

#### Individual Study Results (Seven Studies)

1. Wyman et al. (2014): Large and significant reduction in alcohol use disorder scores pre- to post-intervention (in the IG only). In addition, 73.8% of the IG stated that they reduced or stopped alcohol use before surgery and 64.8% up-to follow-up. Though 9.1 and 17% reported no change in their alcohol use before surgery and up-to follow-up. Zero percent of participants said they would stop using alcohol in the next 6 months, but 18.8% said they would reduce their use. In contrast, 5.9% said they would increase use, and 24% continue to use the same amount. Many participants reported that the questions were not applicable to them.
2. Kummel et al. (2008): Small beneficial effect for the IG over CG at 3 months, but benefits for the CG over IG at 6 and 12 months, with an overall small and non-significant effect size favoring controls—in relation to the between groups proportion of patients reporting zero alcohol use, vs. use more than once a week.
3. McHugh et al. (2001): Non-significant, but moderate (medium-sized effect) reduction in grams per week of alcohol consumed in the IG vs. CG from baseline to 15-months follow-up.
4. Shourie et al. (2006): Moderate but non-significant reduction in alcohol use disorder scores in the IG over CG from baseline to 6-months follow-up. Small and non-significant reduction in grams of alcohol consumed per day in the CG over IG from baseline to follow-up. Non-significantly fewer alcohol-dependent diagnosed CG patients between groups at follow-up, with a small effect.
5. Tønnesen et al. (1999): Moderately, but non-significantly, more non-hazardous alcohol users in the IG vs. CG at 1-month follow-up. Significantly fewer alcohol drinks consumed weekly in the IG vs. CG at follow-up, with a large effect.
6. Tønnesen (2002): Very large and marginally significant benefit for the IG over CG in regard to non-hazardous drinking at 1-month follow-up. Significantly fewer grams per week of alcohol consumed in the IG vs. CG at follow-up, with a large effect—but not at 3-months follow-up, though the effect was moderately large.
7. Weinrieb et al. (2011): Up-to 108 weeks post-randomization the IG consumed significantly fewer drinks, had significantly fewer drinking days and consumed significantly fewer drinks per drinking day vs. the CG, all with very large effect sizes (but with wide confidence intervals). Fewer patients in the CG drank before surgery vs. the IG, though the effect was small and non-significant.

#### Meta-Analysis

A pooled analysis including all six studies (Tønnesen et al., 1999; McHugh et al., 2001; Tønnesen, 2002; Shourie et al., 2006; Kummel et al., 2008; Weinrieb et al., 2011) with between groups alcohol outcomes significantly favored IGs, with a small to moderate effect size (*d*: 0.34; 95% CI: 0.05–0.64; *p* = 0.02). There was significant heterogeneity [*I*^2^ = 66%; *Q*_(5)_ = 14.7, *p* = 0.01].

Separate pooled analyses were conducted for four trials using behavioral interventions (McHugh et al., 2001; Shourie et al., 2006; Kummel et al., 2008; Weinrieb et al., 2011), and the two trials (Tønnesen et al., 1999; Tønnesen, 2002) using disulfiram (as reported previously in Oppedal et al., 2012). Because two of the behavioral-interventions i. did not have alcohol use related inclusion criteria and ii. targeted multiple health behaviors, we decided to conduct a pooled analysis without these studies also.

Across behavioral intervention trials there was a very small, non-significant effect on alcohol consumption [*d*: 0.14; 95% CI: −0.13–0.41; *p* = 0.30; *I*^2^ = 45%; *Q*_(3)_ = 5.5, *p* = 0.14]. In the two behavioral trials with alcohol-related inclusion criteria there was a moderate, but again, highly non-significant effect [*d*: 0.45; 95% CI: −0.49–1.39; *p* = 0.94; *I*^2^ = 70%; *Q*_(1)_ = 3.3, *p* = 0.07]. In the two disulfiram trials there was a large significant effect (*d*: 0.71; 95% CI: 0.36–1.07; *p* < 0.001) with little heterogeneity [*I*^2^ = 0%; *Q*_(1)_ = 0.22, *p* = 0.64].

### Other Recreational Substance Use (ORSU)

#### Individual Study Results (Two Studies)

Only two studies considered ORSU. One study (Wyman et al., 2014) explicitly aimed the intervention at other recreational substance as well as alcohol use, another study (Weinrieb et al., 2011) assessed ORSU in addition to alcohol use, but it was unclear whether this was an intervention target.

1. Wyman et al. (2014): Large and significant reduction in the number of patients reporting ORSU in the last 6 months at follow-up (3-months post-surgery) vs. baseline (IG only). Laboratory screening on the day of surgery found fewer patients in the IG positive for ORSU vs. the CG (11 of the IG [105 patients], 5 controls [56 patients]), though the effect was small and non-significant.
2. Weinrieb et al. (2011): No significant difference between groups in regard to the number of patients using illicit substances post-baseline (four of the IG [46 patients] and three of the CG [45 patients]). There was a small effect favoring the CG.

#### Meta-Analysis

After combining data from between-subjects outcomes in these two studies, the pooled effect size was small, non-significant and favored the IG [*d*: 0.12; 95% CI: −0.24–0.47; *p* = 0.52; *I*^2^ = 0%; *Q*_(1)_ = 0.47, *p* = 0.49].

### Psychological Determinants of Behavior

#### Individual Study Results (Two Studies)

Two studies assessed determinants of perioperative substance use (see Table 2).

1. Ashton et al. (2013): Large pre-post intervention increases in patients' knowledge of the deleterious effects of substance use *per se*; the number of patients reporting that they were “not likely” to return to drinking post-surgery; the number of coping strategies patients could list to enable continuing abstinence; the number of patients citing health reasons as their primary motivation for abstinence.
2. Weinrieb et al. (2011): IG significantly decreased in *ambivalence* and *recognition* scores across time vs. the CG—though, scores *regarding taking steps* did not show a change across time. All three determinants relate to the likelihood of engaging in and seeking out alcohol use treatment.

### Perioperative Complications

#### Individual Study Results (Five Studies)

1. Tønnesen et al. (1999): Significantly more CG patients suffered any complication vs. those in the IG up-to 1 month postoperatively, with a very large effect.
2. Tønnesen (2002): Moderate to large benefit for the IG over CG in relation to total complications up-to 1 month postoperatively, but no significant difference between groups.
3. Wyman et al. (2014): No significant difference in total complications between groups during their hospital stay. Non-significantly fewer cancellations or postponements in surgery in the IG vs. CG (small effect). Non-significantly more perioperative ICU admissions in the IG vs. CG (very small effect).
4. Hansen et al. (2012): No significant difference, but moderate effect favoring the IG in total complications between-groups up-to 3-months follow-up. Non-significantly fewer hospital readmissions in the IG vs. CG up-to 3-months follow-up with a small effect. Significantly more patients in the CG vs. IG met hospital discharge criteria late (moderate to large effect). Non-significantly more “*unintended patient pathways*” (see Table 2) in the CG vs. IG, with a moderate effect.
5. Shourie et al. (2006): Significantly higher total number of complications in the IG vs. CG up-to 5 days postoperatively, with a moderately large effect. Non-significantly more hospital admissions in the CG vs. IG in the previous 6 months with a small to moderate effect (at 6-months follow-up).

#### Meta-Analysis

In a pooled analysis of all five studies assessing perioperative complications, there was a non-significant effect favoring IGs, with a small effect (*d*: 0.24; 95% CI: −0.26–0.73; *p* = 0.35). There was significant heterogeneity [*I*^2^ = 74%; *Q*_(4)_ = 12.3, *p* = 0.004]. In total there were sixty-two complications in the IGs (253 patients) and 67 in the CGs (235 patients).

A pooled analysis excluding Hansen et al. (2012) was conducted for two reasons: i. the authors aimed to target a number of health behaviors as well as alcohol (confounding the potential beneficial effect of preoperative alcohol reduction), and ii. because the authors only recruited four participants they deemed hazardous alcohol users. The effect was again non-significant and small [*d*: 0.21; 95% CI: −0.40–0.82; *p* = 0.50; *I*^2^ = 78%; *Q*_(3)_ = 13.8, *p* = 0.003].

Lastly, analyses were conducted separately (without Hansen et al., 2012) for the two behavioral-intervention controlled trials (Shourie et al., 2006; Wyman et al., 2014) and the two disulfiram-based randomized controlled trials (Tønnesen et al., 1999; Tønnesen, 2002) (as reported previously in Oppedal et al., 2012). The latter found a large significant effect [*d*: 0.71; 95% CI: 0.36–1.07; *p* < 0.001; *I*^2^ = 0%; *Q*_(1)_ = 0.2, *p* = 0.64], the former a small non-significant effect [*d*: −0.23; 95% CI: −0.71–0.24; *p* = 0.33; *I*^2^ = 61%; *Q*_(1)_ = 2.6, *p* = 0.11].

### Length of Hospital Stay

#### Individual Study Results (Four Studies)

1. Tønnesen et al. (1999): Zero difference in length of stay.
2. Tønnesen (2002): Zero difference in length of stay.
3. Shourie et al. (2006): Non-significantly fewer days in the IG vs. CG with a small effect (a mean reduction of 1 day).
4. Hansen et al. (2012): Significantly fewer days in the IG vs. CG with a moderate effect (a median reduction of 1 day).

#### Meta-Analysis

A pooled analysis of all four studies significantly favored IGs, with a small effect size [*d*: 0.27; 95% CI: 0.04–0.5; *p* = 0.02]. There was low heterogeneity [*I*^2^ = 5%; *Q*_(3)_ = 3.2, *p* = 0.37].

Again, a pooled analysis was conducted without Hansen et al. (2012), this resulted in a small and non-significant effect [*d*: 0.12; 95% CI: −0.16–0.41; *p* = 0.41; *I*^2^ = 0%; *Q*_(2)_ = 0.4, *p* = 0.82].

### Mortality

#### Individual Study Results (Five Studies)

1. Tønnesen et al. (1999): One death occurred in the IG, and two in the CG up-to 1-month follow-up—a non-significant, moderate effect favoring the IG.
2. Tønnesen (2002): One death occurred in the total sample (CG) up-to 1 month follow-up—a non-significant, moderate to large effect favoring the IG.
3. Shourie et al. (2006): One death occurred in each group up-to 5 days post-surgery—a non-significant, small to moderate effect favoring the CG.
4. Hansen et al. (2012): Zero deaths per group up-to 3-months follow-up.
5. Rideout et al. (2012): Up-to 12 years post-randomization in McHugh et al. (2001), there were 15 (50 patients) and 26 (60 patients) deaths in the IG and CG, respectively—a non-significant, small effect favoring the IG (likely due to general, not perioperative benefits of alcohol use reduction).

#### Meta-Analysis

When considering perioperative mortality post-surgery (three studies targeting alcohol use only: (Tønnesen et al., 1999; Tønnesen, 2002; Shourie et al., 2006) the effect size was small and non-significant (*d*: 0.21; 95% CI: −0.69–1.10; *p* = 0.65) with little heterogeneity [*I*^2^ = 0%; *Q*_(2)_ = 0.96, *p* = 0.62]. Across the three studies there were two deaths in the IGs (126 patients), and in the CGs four (79 patients). An additional study was not included in this analysis as zero deaths were reported in either group up to 3 months post-surgery (Hansen et al., 2012).

The two disulfiram trials were pooled in a separate analysis. Again, as reported previously in Oppedal et al. (2012), there was a non-significant, moderately large benefit for IG patients with low heterogeneity [*d*: 0.51; 95% CI: −0.59–1.60; *p* = 0.36; *I*^2^ = 0%; *Q*_(1)_ = 0.1, *p* = 0.77]. There were three deaths in the CGs (34 patients) and one death in the IGs (35 patients) up to 1-month post-surgery.

### Health Status/Health Related Quality of Life

#### Individual Study Results (Five Studies)

Five studies (McHugh et al., 2001; Shourie et al., 2006; Kummel et al., 2008; Weinrieb et al., 2011; Hansen et al., 2012) assessed general health related outcomes.

1. Hansen et al. (2012): Small non-significant improvement in health-related quality of life in the IG vs. CG from baseline to 3-months follow-up. Likewise for disease specific outcome scores.
2. McHugh et al. (2001): Average effect size across health-related domains significantly much improved in the IG over CG from baseline to 15-months follow-up. Large and significant benefit for the IG in terms of both depression and anxiety scores from baseline to follow-up. A range of physical outcomes were improved in the IG vs. CG, including BMI, and blood pressure from baseline to follow-up.
3. Shourie et al. (2006): Small, non-significant benefits for the IG in regard to the number of GP visits, and number of days off work sick in the last 6 months, at 6-month follow-up.
4. Weinrieb et al. (2011): The authors report that there were no significant differences across all time points for both groups in relation to health-related quality of life and very little difference in regard to depression and anxiety. Effect sizes could not be calculated.
5. Kummel et al. (2008): Average effect size for symptoms of angina pectoris (not at all vs. at least once daily) across all time points was very small and non-significantly favored the CG. Across all time points, the IG reported significantly, but moderately, better functional abilities vs. controls.

### Patient Satisfaction

#### Individual Study Results (Three Studies)

Of three studies (McHugh et al., 2001; Ashton et al., 2013; Wyman et al., 2014) assessing patient satisfaction, all reported high patient satisfaction with the respective interventions.

## Discussion

The present review primarily aimed to unite and update two previous reviews (Oppedal et al., 2012; Fernandez et al., 2015) of interventions for preoperative alcohol use, and for the first time, systematically review the literature on preoperative interventions for other recreational substance use (ORSU). Identified were a small number of low-powered, high risk of bias and demographically and methodologically heterogeneous studies. Based on current evidence it cannot be concluded that interventions may be delivered prior to patients' surgery to reduce ORSU, and certainly no one intervention strategy can be recommended. Across individual studies, there was some evidence that interventions can be delivered from pre- to post-surgery to reduce alcohol consumption, though two small trials using disulfiram (Tønnesen et al., 1999; Tønnesen, 2002) were far superior to trials of behavioral strategies alone. Given small effects on alcohol consumption, there was no evidence that preoperative interventions for alcohol or ORSU could reduce the incidence of perioperative complications on the whole, though again, Tønnesen et al. (1999) and Tønnesen (2002) bucked this trend. Lastly, there were no clear effects of interventions on mortality and length of hospital stay in studies solely targeting alcohol use. Results, review limitations and recommendations are respectively discussed henceforth.

### Summary of Results

A major finding of this review was the lack of studies investigating interventions for preoperative ORSU, despite evidence that ORSU can impact on surgical outcomes. Wyman et al. (2014) targeted ORSU (in addition to alcohol use), and Weinrieb et al. (2011) assessed ORSU, but it was unclear whether ORSU was targeted. While the former found that IG patients were less likely to report using an illicit substance at follow-up vs. baseline (with a very large effect), the absence of controls obfuscates any causal relation the intervention may have had on ORSU. Furthermore, responses were susceptible to social desirability biases given that a non-validated, non-blinded self-report measure was used—clearly evidenced by the fact that whilst the study did find that more controls were positive for ORSU via objective screening on the day of surgery, the effect was very small. In this study, over 40% of patients had previously entered substance use treatment, suggesting that patients were either current or previously dependent recreational substance users. ORSU dependency is notoriously difficult to treat, especially in the case of opiates—speculatively, this short, one session intervention may have been more effective with more casual users. More intensive strategies may be necessary for those with dependency. Again, while Weinrieb et al. (2011) found that controls were less likely than intervention patients to be positive for ORSU, baseline ORSU data was absent from the final publication and could not be retrieved after correspondence with the authors. Given that the study primarily aimed to reduce alcohol use this is unsurprising. Pooling both study's between-group outcomes found a small and non-significant benefit for the IGs, but more evidence is needed to form substantial conclusions regarding the efficacy of ORSU targeted interventions.

While there is more evidence to suggest interventions can be delivered to reduce preoperative alcohol vs. ORSU, the evidence is still not strong. A pooled analysis of all six studies investigating this outcome has a small and significant effect, though the confidence intervals were wide and studies heterogeneous. Behavioral interventions showed to be largely ineffective on the whole. Two behavioral intervention trials (Shourie et al., 2006; Weinrieb et al., 2011) which included risky alcohol users fared better, but this was to be expected given that these interventions had primary a focus on alcohol use (as opposed to multiple behaviors). Disulfiram trials (Tønnesen et al., 1999; Tønnesen, 2002) were by far the most effective—both achieving complete abstinence prior to surgery, and benefits up to 1 month post-surgery. However, these trials were both small, and given dangerous side effects (e.g., neurotoxicity) and generally low compliance (e.g., Fuller et al., 1986; Pettinati et al., 2008; Kalra et al., 2014), disulfiram is a medication to be used with caution and only in a small population of highly motivated, alcohol dependent patients. There are however a number of other pharmaceutical protocols, shown useful in similar contexts, which may aid detoxification in the preoperative context, such as those involving pregabalin and clomethiazole (Di Nicola et al., 2010; e.g., Sychla et al., 2017).

As there were no significant overall effects on perioperative complications, mortality and length of hospital stay, this may be explained by intervention timing. Some studies neglected to report the time from intervention to surgery, thus interventions may have been delivered so close to surgery that any beneficial effects could not have time to materialize. Furthermore, acute cessation in risky alcohol users may incur withdrawal symptoms, leading to counterproductive effects. In support, Shourie et al. (2006), who did report time to surgery, and included risky alcohol users only, found significantly more complications in the IG, but had only a minimum of 1 week from intervention to surgery. While two trials of disulfiram (Tønnesen et al., 1999; Tønnesen, 2002) appeared superior to two behavioral trials for perioperative complications, this may be explained by length of follow-up. While Tønnesen et al. (1999) and Tønnesen (2002)) assessed between group complications up to 1 month post-surgery, Wyman et al. (2014) and Shourie et al. (2006) only assessed intraoperative and immediate post-surgical complications. It is well-known that many complications may only emerge in the postoperative recovery period, thus these studies may have missed any between group differences in recovery. Lack of complications in the immediate postoperative period may also explain lack of differences in length of hospital stay across all studies—and as is likely in the case of mortality, may be a result of small sample sizes, and thus lack of power. Lastly, the fact that the disulfiram trials both achieved complete preoperative abstinence as opposed to be moderate reductions in alcohol use in both behavioral trials, more than likely had beneficial implications for surgical complications.

One study (Ashton et al., 2013) showed that an intervention could boost patients' intentions to reduce alcohol-use post-surgery, increase their knowledge of the harms of perioperative substance use, and increase the number of coping strategies—to aid perioperative cessation—patients could list. Though it is unclear whether this influenced their actual behavior, medium to large effects on intentions may lead to small to medium effects on behavior (Gollwitzer and Sheeran, 2006). Increased knowledge of harm *may* be associated with lower alcohol consumption also (e.g., Thadani et al., 2009). Another study (Weinrieb et al., 2011) found showed some beneficial intervention effects on stages of change scores. This is a positive finding as motivation to reduce alcohol use is a predictor of an actual reduction in alcohol use (e.g., Kohler and Hofmann, 2015). There were several methodological issues with these studies however, as both studies were generally at high risk-of-bias.

There were some positive intervention effects regarding other outcomes. One study (McHugh et al., 2001) found intervention benefits for BMI/cholesterol and general health, others quality of life. Though all of these results may be confounded by targeting of multiple health behaviors, another, exclusively alcohol-focussed study (Weinrieb et al., 2011), found intervention benefits for outcomes relating to general health.

All interventions that assessed patient satisfaction reported high levels. Patients therefore may be receptive to behavior change in the perioperative period. This may have positive implications for patient retention within trials and may foster patient acceptability and motivation. However, almost uniformly high patient satisfaction may highlight another issue; those recruited may represent only those most motivated, and thus intervention efficacy may be artificially inflated. Indeed, it took many studies over a year to recruit their participants, and those agreeing may have been only those most receptive to intervention. This is highlighted by an incredibly small proportion (1.5%) of those approached being recruited in Tønnesen (2002). Recruitment is clearly a challenge in this area, with a relatively small total *n* of 903 across nine trials.

### Review Limitations

The relative efficacy of behavioral interventions on preoperative vs. postoperative alcohol use (or ORSU) use could not be established—an important distinction, as complications, such as prolonged bleeding time may be reversed by a preoperative alcohol abstinence period of 4 weeks. Just one behavioral-only intervention (Weinrieb et al., 2011) Assessed post-baseline, but preoperative alcohol use, but did not assess complications—so the causal relation between preoperative reduction or abstinence and complications could not be delineated. As few studies were identified, meta-regression analyses aiming to assess whether retrospectively identified BCTs could predict intervention efficacy could not be performed.

Across studies, no pre-published study protocols were found, thus there was a risk of selective reporting; just five studies were randomized, though just two (Tønnesen et al., 1999; Tønnesen, 2002) reported the method of sequence generation; no studies were blinded in any way; and just two studies (again, Tønnesen et al., 1999; Tønnesen, 2002) concealed allocations. Many studies had a large bias toward male patients, and one study (Kummel et al., 2008) included patients over 65 only. Such biases may have influenced the effect sizes across studies, so must be considered when interpreting the results. Additionally, different surgical populations have different prevalence rates of substance use and different underlying conditions and procedures naturally lead to different complication rates. Readers should be mindful that the type of surgery may have impacted on both substance use cessation rates and the number of complications suffered.

There were a number of other potential moderators, such as length of follow-up, and patient demographic variables (e.g., comorbidities). If more studies were available, subgroup analyses would have been useful to investigate these. Publication bias might account for some of the observed effects also. The number of studies located was not sufficient to conduct a formal analysis of this phenomenon.

### Recommendations

For behavioral interventions, just one study cited a replicable intervention protocol. For most studies, it was not explicit what intervention techniques were used. Therefore, accurately replicating successful interventions is difficult for future researchers. In this review, behavior change techniques (BCTs) were respectively identified—providing a non-comprehensive indication of the techniques used, but the above issues made the process difficult. Future studies should aim to characterize BCTs, or at minimum, cite—or describe in sufficient detail—intervention protocols. Further, it is not clear whether any studies undertook appropriate elicitation research—that is, systematically identified salient, population-specific behavioral determinants. This is a vital component of intervention development, ensuring that intervention techniques are aimed at appropriate targets and allowing researchers to link interventions with behavior change theory (identifying interventions' *mechanisms of action*). Numerous guidelines exist (e.g., Michie et al., 2011) for this process and researchers should be mindful of these conventions.

Future studies should consider solely targeting alcohol or ORSU, so that any confounding influence of changing other behaviors on perioperative outcomes is minimized. Researchers should also consider intervening on *specific* recreational substances (an intervention may be useful at reducing cannabis, but not heroin use). Researchers should aim to measure behavior both preoperatively and postoperatively, to (i) assess whether interventions can have differential effects between time periods, and (ii) assess the causal relation between a preoperative reduction in substance use and perioperative outcomes. The number and length of intervention sessions, and the time from intervention to surgery, should be reported also, as these may be key moderators of intervention efficacy. Researchers may also explore whether intervention efficacy is moderated by patients' surgical procedure, and the frequency and/or extent of patients' alcohol or ORSU. This may be achieved by having inclusion criteria that limits the sample to those undergoing specific surgeries, and those having varying levels of substance use.

Given their diversity in this review, it would be useful for researchers to standardize outcome measures, easing comparisons between studies. Standardization may be achieved by developing a substance-use specific core outcome set (see the COMET initiative; Prinsen et al., 2014), alongside the current set for postoperative complications generally (Jammer et al., 2015). It would also be beneficial to include more patient-centered outcomes, such as return to work, functioning and quality of life. Similarly, objective measures for substance use are needed (e.g., urine and breath tests) to reduce recall and social desirability biases.

Future studies should aim to minimize risk-of-bias and improve on their designs. Though blinding is difficult with behavioral interventions, blinding of assessors could be achieved. Additionally, future studies should use randomized designs with appropriate controls, report their random sequence-generation method and pre-publish their protocols. Future studies should provide formal power calculations to estimate required sample sizes before recruitment, and therefore employ larger sample sizes to identify between-group differences in somewhat rare surgical complications. Studies should recruit more representative samples, including a wider range of patient ages, ethnicities and an equal distribution of both sexes.

### Conclusion

Despite limitations, we have identified important gaps in the evidence base, Such as the small number of trials to reduce ORSU versus alcohol use. Given the efficacy of the two small disulfiram trials, prescribing this pharmaceutical may be useful for a small subset of patients with alcohol dependency, though it may only be appropriate for patients most at risk. For non-dependent heavy alcohol users, the best strategy has yet to be identified—though motivational strategies, and strategies targeted specifically at alcohol use, marginally appear to be useful. Future studies would benefit from higher quality designs and more standardization in measures and inclusion criteria across the field. In the case of behavioral interventions, more intervention development work may be necessary. As more robust evidence accumulates, more substantial recommendations for best practice may be drawn.

## Author Contributions

LB, AP, IK, RL, and AK: conceptualization; LB, AP, IK, and RL: formal analysis, methodology, and investigation; LB: writing—original draft preparation; LB, AP, RL, AK, and IK: writing—review and editing.

### Conflict of Interest Statement

The authors declare that the research was conducted in the absence of any commercial or financial relationships that could be construed as a potential conflict of interest.
