# Supplementary material for: Preoperative Interventions for Alcohol and Other Recreational Substance Use: A Systematic Review and Meta-Analysis
Source: Front Psychol. 2019 Feb 4;10:34. doi: 10.3389/fpsyg.2019.00034 (PMC6369879; doi:10.3389/fpsyg.2019.00034)
Supplement: Supplementary file 1 [file Data_Sheet_1.docx]

**Appendix 1. Search Strategy**

**EMBASE**

1. EXP REVIEW/
2. (LITERATURE ADJ3 REVIEW$).TI,AB.
3. EXP META ANALYSIS/
4. EXP "SYSTEMATIC REVIEW"/

## 5. OR/1-4

1. (MEDLINE OR MEDLARS OR EMBASE OR PUBMED OR CINAHL OR AMEDs OR PSYCHLIT OR PSYCLIT OR PSYCHINFO OR PSYCINFO OR SCISEARCH OR COCHRANE).TI,AB.
2. RETRACTED ARTICLE/

## 8. 6 OR 7

## 9. 5 AND 8

1. (SYSTEMATIC$ ADJ2 (REVIEW$ OR OVERVIEW)).TI,AB.
2. (META?ANAL$ OR META ANAL$ OR META-ANAL$ OR METAANAL$ OR METANAL$).TI,AB.
3. **9 OR 10 OR 11**
4. (RANDOM$ OR PLACEBO$ OR SINGLE BLIND$ OR DOUBLE BLIND$ OR TRIPLE BLIND$).TI,AB.
5. RETRACTED ARTICLE/

## 15. OR/13-14

1. (ANIMAL$ NOT HUMAN$).SH,HW.
2. (BOOK OR CONFERENCE PAPER OR EDITORIAL OR LETTER OR REVIEW).PT.NOT EXP RANDOMIZED CONTROLLED TRIAL/
3. (RANDOM SAMPL$ OR RANDOM DIGIT$ OR RANDOM EFFECT$ OR RANDOM SURVEY OR RANDOM REGRESSION).TI,AB. NOT EXP RANDOMIZED CONTROLLED TRIAL/

## 19. 15 NOT (16 OR 17 OR 18)

1. EXP COHORT ANALYSIS/
2. EXP LONGITUDINAL STUDY/
3. EXP PROSPECTIVE STUDY/
4. EXP FOLLOW UP/
5. COHORT$.TW.
6. EXP CASE CONTROL STUDY/
7. (CASE$ AND CONTROL$).TW.
8. EXP CASE STUDY/
9. (CASE$ AND SERIES).TW.
10. CASE REPORT/
11. (CASE$ ADJ2 REPORT$).TW.
12. (CASE$ ADJ2 STUD$).TW.

## 32. OR/20-31

1. EXP DRINKING BEHAVIOR/
2. EXP ALCOHOLISM/
3. ALCOHOL-RELATED DISORDER*.MP.
4. (DRINK* ADJ3 (BEHAVIOUR OR HAZARDOUS OR HARMFUL* OR DEPENDENCE)).MP.
5. (ALCOHOL* ADJ3 (DRINK* OR USE* OR INTAKE OR INTERVENTION* OR EDUCATION OR PROGRAM* OR ABUSE$ OR MISUSE$ OR PROBLEM$ OR DEPEND$ OR ADDICT$ OR DISORDER$)).MP.
6. DRUG DEPENDENCE/ OR EXP ADDICTION/ OR EXP BENZODIAZEPINE DEPENDENCE/ OR EXP CANNABIS ADDICTION/ OR EXP COCAINE DEPENDENCE/ OR EXP CONGENITAL DRUG DEPENDENCE/ OR EXP DRUG ABUSE PATTERN/ OR EXP DRUG MISUSE/ OR EXP DRUG SEEKING BEHAVIOR/ OR EXP GLUE SNIFFING/ OR EXP METHAMPHETAMINE DEPENDENCE/ OR EXP MULTIPLE DRUG ABUSE/ OR EXP NARCOTIC DEPENDENCE/
7. (DRUG ABUSE$ OR DRUG MISUSE$ OR DRUG PROBLEM$ OR DRUG DEPEND$ OR DRUG ADDICT$ OR DRUG DISORDER$ OR ILLICIT DRUG$).TI,AB.
8. ((NARCOTIC$ OR HEROIN OR OPIATE$ OR OPIOID$ OR OPIUM OR COCAINE$ OR CANNABIS$ OR MARIJUANA OR MARIHUANA OR HASHISH OR PHENCYCLIDINE OR BENZODIAZ$ OR BARBITURATE$ OR AMPHETAMINE$ OR MDMA OR HALLUCINOGEN$ OR KETAMINE OR LSD OR INHALANT$ OR SUBSTANCE$) ADJ3 (ABUSE$ OR MISUSE$ OR USE$ OR PROBLEM$ OR DEPEND$ OR ADDICT$ OR DISORDER$)).TI,AB.

## 41. 33 OR 34 OR 35 OR 36 OR 37 OR 38 OR 39 OR 40

1. EXP POSTOPERATIVE COMPLICATION/
2. EXP PREOPERATIVE CARE/ OR EXP PREOPERATIVE EDUCATION/ OR EXP PREOPERATIVE EVALUATION/
3. SURGERY/
4. (SURGICAL ADJ3 (SETTING* OR PATIENT* OR OUTCOME*)).MP.
5. (PREOP* ADJ3 (INTERVENTION* OR EDUCATION OR PROGRAM* OR STUD* OR TREATMENT* OR PREVENT* OR THER* OR SERV* OR COUNSEL* OR SUPP* OR INFO* OR CONSULT* OR CARE)).MP.
6. (PRE-OP*ADJ3 (INTERVENTION* OR EDUCATION OR PROGRAM* OR STUD* OR TREATMENT* OR PREVENT* OR THER* OR SERV* OR COUNSEL* OR SUPP* OR INFO* OR CONSULT* OR CARE)).MP.
7. (PERIOP* ADJ3 (INTERVENTION* OR EDUCATION OR PROGRAM* OR STUD* OR TREATMENT* OR PREVENT* OR THER* OR SERV* OR COUNSEL* OR SUPP* OR INFO* OR CONSULT* OR CARE)).MP.
8. (PERI-OP* ADJ3 (INTERVENTION* OR EDUCATION OR PROGRAM* OR STUD*

OR TREATMENT* OR PREVENT* OR THER* OR SERV* OR COUNSEL* OR SUPP* OR INFO* OR CONSULT* OR CARE)).MP.

## 50. 42 OR 43 OR 44 OR 45 OR 46 OR 47 OR 48 OR 49

## 51. 12 OR 19 OR 32

## 52. 51 AND 50 AND 41

**MEDLINE**

1. (REVIEW OR REVIEW,TUTORIAL OR REVIEW, ACADEMIC).PT.
2. (MEDLINE OR MEDLARS OR EMBASE OR PUBMED OR COCHRANE).TW,SH.
3. (SCISEARCH OR PSYCHINFO OR PSYCINFO).TW,SH.
4. (PSYCHLIT OR PSYCLIT).TW,SH.
5. CINAHL.TW,SH.
6. ((HAND ADJ2 SEARCH$) OR (MANUAL$ ADJ2 SEARCH$)).TW,SH.
7. (ELECTRONIC DATABASE$ OR BIBLIOGRAPHIC DATABASE$ OR COMPUTERI?ED DATABASE$ OR ONLINE DATABASE$).TW,SH.
8. (POOLING OR POOLED OR MANTEL HAENSZEL).TW,SH.
9. (PETO OR DERSIMONIAN OR DER SIMONIAN OR FIXED EFFECT).TW,SH.
10. (RETRACTION OF PUBLICATION OR RETRACTED PUBLICATION).PT.

## 11. OR/2-10

## 12. 1 AND 11

1. META-ANALYSIS.PT.
2. META-ANALYSIS.SH.
3. (META-ANALYS$ OR META ANALYS$ OR METAANALYS$).TW,SH.
4. (SYSTEMATIC$ ADJ5 REVIEW$).TW,SH.
5. (SYSTEMATIC$ ADJ5 OVERVIEW$).TW,SH.
6. (QUANTITATIV$ ADJ5 REVIEW$).TW,SH.
7. (QUANTITATIV$ ADJ5 OVERVIEW$).TW,SH.
8. (QUANTITATIV$ ADJ5 SYNTHESIS$).TW,SH.
9. (METHODOLOGIC$ ADJ5 REVIEW$).TW,SH.
10. (METHODOLOGIC$ ADJ5 OVERVIEW$).TW,SH.
11. (INTEGRATIVE RESEARCH REVIEW$ OR RESEARCH INTEGRATION).TW.

## 24. OR/13-23

## 25. 12 OR 24

1. "RANDOMIZED CONTROLLED

TRIAL".PT.

1. (RANDOM$ OR PLACEBO$ OR SINGLE BLIND$ OR DOUBLE BLIND$ OR TRIPLE BLIND$).TI,AB.
2. (RETRACTION OF PUBLICATION OR RETRACTED PUBLICATION).PT.

## 29. OR/26-28

1. (ANIMALS NOT HUMANS).SH.
2. ((COMMENT OR EDITORIAL OR METAANALYSIS OR PRACTICE-GUIDELINE OR REVIEW OR LETTER OR JOURNAL CORRESPONDENCE) NOT "RANDOMIZED CONTROLLED TRIAL").PT.
3. (RANDOM SAMPL$ OR RANDOM DIGIT$ OR RANDOM EFFECT$ OR RANDOM SURVEY OR RANDOM REGRESSION).TI,AB. NOT "RANDOMIZED CONTROLLED TRIAL".PT.

## 33. 29 NOT (30 OR 31 OR 32)

1. EXP COHORT STUDIES/
2. COHORT$.TW.
3. CONTROLLED CLINICAL TRIAL.PT.
4. EPIDEMIOLOGIC METHODS/
5. EXP CASE-CONTROL STUDIES/
6. (CASE$ AND CONTROL$).TW.
7. (CASE$ AND SERIES).TW.
8. CASE REPORTS.PT.
9. (CASE$ ADJ2 REPORT$).TW.
10. (CASE$ ADJ2 STUD$).TW.

## 44. OR/34-43

1. ALCOHOL-RELATED DISORDERS/ OR EXP ALCOHOLIC INTOXICATION/ OR EXP ALCOHOLISM/ OR EXP BINGE DRINKING/
2. (ALCOHOL* ADJ3 (DRINK* OR USE* OR INTAKE OR INTERVENTION* OR EDUCATION OR PROGRAM* OR ABUSE$ OR MISUSE$ OR PROBLEM$ OR DEPEND$ OR ADDICT$ OR DISORDER$)).MP.
3. (DRINK* ADJ3 (BEHAVIOUR OR HAZARDOUS OR HARMFUL* OR DEPEND$ OR ADDICT$ OR OVER$ OR PROBLEM$ OR INTERVENTION$ OR EDUCATION OR PROGRAM)).MP.
4. SUBSTANCE-RELATED DISORDERS/ OR EXP AMPHETAMINE-RELATED DISORDERS/ OR EXP COCAINERELATED DISORDERS/ OR EXP INHALANT ABUSE/ OR EXP MARIJUANA ABUSE/ OR EXP OPIOID-RELATED DISORDERS/ OR EXP PHENCYCLIDINE ABUSE/ OR EXP SUBSTANCE ABUSE, INTRAVENOUS/ OR EXP SUBSTANCE WITHDRAWAL SYNDROME/
5. DRUG USERS/
6. ((NARCOTIC$ OR HEROIN OR OPIATE$ OR OPIOID$ OR OPIUM OR COCAINE$ OR CANNABIS$ OR MARIJUANA OR MARIHUANA OR HASHISH OR PHENCYCLIDINE OR BENZODIAZ$ OR BARBITURATE$ OR AMPHETAMINE$ OR MDMA OR HALLUCINOGEN$ OR KETAMINE OR LSD OR INHALANT$ OR SUBSTANCE$) ADJ3 (ABUSE$ OR MISUSE$ OR USE$ OR PROBLEM$ OR DEPEND$ OR ADDICT$ OR DISORDER$)).TI,AB.
7. (DRUG ABUSE$ OR DRUG MISUSE$ OR DRUG PROBLEM$ OR DRUG DEPEND$ OR DRUG ADDICT$ OR DRUG DISORDER$ OR ILLICIT DRUG$).TI,AB.

## 52. OR/45-51

1. EXP POSTOPERATIVE COMPLICATIONS/
2. EXP PERIOPERATIVE CARE/ OR EXP PREOPERATIVE CARE/
3. SURGICAL PROCEDURES, OPERATIVE/
4. (SURGICAL ADJ3 (SETTING* OR PATIENT* OR OUTCOME*)).MP.
5. (PREOP* ADJ3 (INTERVENTION* OR EDUCATION OR PROGRAM* OR STUD*OR TREATMENT* OR PREVENT* OR THER* OR SERV* OR COUNSEL* OR SUPP* OR INFO* OR CONSULT* OR CARE)).MP.
6. (PRE-OP*ADJ3 (INTERVENTION* OR EDUCATION OR PROGRAM* OR STUD*OR TREATMENT* OR PREVENT* OR THER* OR SERV* OR COUNSEL* OR SUPP* OR INFO* OR CONSULT* OR CARE)).MP.
7. (PERIOP* ADJ3 (INTERVENTION* OR EDUCATION OR PROGRAM* OR STUD*OR TREATMENT* OR PREVENT* OR THER* OR SERV* OR COUNSEL* OR SUPP* OR INFO* OR CONSULT* OR CARE)).MP.
8. (PERI-OP* ADJ3 (INTERVENTION* OR EDUCATION OR PROGRAM* OR STUD* OR TREATMENT* OR PREVENT* OR THER* OR SERV* OR COUNSEL* OR SUPP* OR INFO* OR CONSULT* OR CARE)).MP.

## 61. OR/53-60

## 62. 25 OR 33 OR 44

## 63. 62 AND 61 AND 52

**PSYCINFO**

1. EXP "LITERATURE REVIEW"/ OR EXP META ANALYSIS/
2. (LITERATURE ADJ3 REVIEW$).TI,AB.
3. (SYSTEMATIC$ ADJ2 (REVIEW$ OR OVERVIEW)).TI,AB.
4. (META?ANAL$ OR META ANAL$ OR META-ANAL$ OR METAANAL$ OR METANAL$).TI,AB.

## 5. 1 OR 2 OR 3 OR 4

1. EXP CLINICAL TRIALS/
2. (RANDOM$ OR PLACEBO$ OR SINGLE BLIND$ OR DOUBLE BLIND$ OR TRIPLE BLIND$).TI,AB.
3. EXP COHORT ANALYSIS/
4. COHORT$.TW.
5. EXP CASE REPORT/
6. (CASE$ ADJ2 STUD$).TW.
7. EXP FOLLOWUP STUDIES/ OR EXP PROSPECTIVE STUDIES/ OR EXP LONGITUDINAL STUDIES/ OR EXP RETROSPECTIVE STUDIES/
8. EXP EXPERIMENTAL DESIGN/
9. EXP TREATMENT EFFECTIVENESS EVALUATION/
10. EXP DRUG THERAPY/

## 16. 6 OR 7 OR 8 OR 9 OR 10 OR 11 OR 12 OR 13 OR 14 OR 15

1. EXP ADDICTION/
2. EXP ALCOHOLISM/ OR EXP ALCOHOL ABUSE/ OR EXP ALCOHOL INTOXICATION/ OR EXP ALCOHOL REHABILITATION/
3. (DRINK* ADJ3 (BEHAVIO?R OR HAZARDOUS OR HARMFUL* OR DEPENDENCE)).MP.
4. (ALCOHOL* ADJ3 (DRINK* OR USE* OR INTAKE OR INTERVENTION* OR EDUCATION OR PROGRAM* OR ABUSE$ OR MISUSE$ OR PROBLEM$ OR DEPEND$ OR ADDICT$ OR DISORDER$)).MP.
5. EXP DRUG ADDICTION/ OR EXP DRUG DEPENDENCY/ OR EXP HEROIN ADDICTION/ OR EXP DRUG ABUSE/ OR EXP DRUG SEEKING/ OR EXP DRUG WITHDRAWAL/ OR EXP INTRAVENOUS DRUG USAGE/ OR EXP METHADONE MAINTENANCE/ OR EXP POLYDRUG ABUSE/ OR EXP DRUG ABSTINENCE/ OR EXP DETOXIFICATION/ OR EXP DRUG REHABILITATION/
6. ((NARCOTIC$ OR HEROIN OR OPIATE$ OR OPIOID$ OR OPIUM OR COCAINE$ OR CANNABIS$ OR MARIJUANA OR MARIHUANA OR HASHISH OR PHENCYCLIDINE OR BENZODIAZ$ OR BARBITURATE$ OR AMPHETAMINE$ OR MDMA OR HALLUCINOGEN$ OR KETAMINE OR LSD OR INHALANT$ OR SUBSTANCE$) ADJ3 (ABUSE$ OR MISUSE$ OR USE$ OR PROBLEM$ OR DEPEND$ OR ADDICT$ OR DISORDER$)).TI,AB.
7. (DRUG ABUSE$ OR DRUG MISUSE$ OR DRUG PROBLEM$ OR DRUG DEPEND$ OR DRUG ADDICT$ OR DRUG DISORDER$ OR ILLICIT DRUG$).TI,AB.

## 24. 17 OR 18 OR 19 OR 20 OR 21 OR 22 OR 23

1. EXP POSTSURGICAL COMPLICATIONS/
2. EXP SURGERY/
3. (SURGICAL ADJ3 (SETTING* OR PATIENT* OR OUTCOME*)).MP.
4. (PREOP* ADJ3 (INTERVENTION* OR EDUCATION OR PROGRAM* OR STUD* OR TREATMENT* OR PREVENT* OR THER* OR SERV* OR COUNSEL* OR SUPP* OR INFO* OR CONSULT* OR CARE)).MP.
5. (PRE-OP*ADJ3 (INTERVENTION* OR EDUCATION OR PROGRAM* OR STUD* OR TREATMENT* OR PREVENT* OR THER* OR SERV* OR COUNSEL* OR SUPP* OR INFO* OR CONSULT* OR CARE)).MP.
6. (PERIOP* ADJ3 (INTERVENTION* OR EDUCATION OR PROGRAM* OR STUD*OR TREATMENT* OR PREVENT* OR THER* OR SERV* OR COUNSEL* OR SUPP* OR INFO* OR CONSULT* OR CARE)).MP.
7. (PERI-OP* ADJ3 (INTERVENTION* OR EDUCATION OR PROGRAM* OR STUD*OR TREATMENT* OR PREVENT* OR THER* OR SERV* OR COUNSEL* OR SUPP* OR INFO* OR CONSULT* OR CARE)).MP.

## 32. 25 OR 26 OR 27 OR 28 OR 29 OR 30 OR 31

## 33. 5 OR 16

## 34. 33 AND 24 AND 32

**CINAHL**

1. MH "SYSTEMATIC REVIEW" or MH "META ANALYSIS" or MH "REVIEW"
2. LITERATURE N3 REVIEW* or SYSTEMATIC N2 REVIEW*
3. META?ANAL* or META ANAL* or METAANAL* or METAANAL* or METANAL*
4. MH "CLINICAL TRIALS+"
5. PT CLINICAL TRIAL or TI CLINIC* N1 TRIAL* or AB CLINIC* N1 TRIAL*
6. MH "RANDOM ASSIGNMENT"
7. TI (SINGL* OR DOUBL* OR TREBL* OR TRIPL*) or TI RANDOMI?ED CONTROL* TRIAL* or AB RANDOMI?ED CONTROL* TRIAL*
8. MH "PROSPECTIVE STUDIES" OR MH "CONCURRENT PROSPECTIVE STUDIES" OR MH "NONCONCURRENT PROSPECTIVE STUDIES" OR MH "PANEL STUDIES" OR MH "POSTEXPOSURE FOLLOW-UP" OR MH "CASE CONTROL STUDIES" OR MH "CASE STUDIES"
9. COHORT*
10. (CASE* AND CONTROL*) OR (CASE* AND SERIES) OR (CASE* N2 REPORT*) OR (CASE* N2 STUD*)

## 11. 1 OR 2 OR 3 OR 4 OR 5 OR 6 OR 7 OR 8 OR 9 OR 10

1. MH "ALCOHOL-RELATED DISORDERS+" OR MH "ALCOHOLISM” OR MH “DRINKING BEHAVIO?R+” OR MH “ALCOHOLIC INTOXICATION”
2. TX (ALCOHOL*) N3 TX (DRINK* OR USE* OR INTAKE OR INTERVENTION* OR EDUCATION OR PROGRAM*)
3. TX (DRINK*) N3 TX (BEHAVIO?R OR HAZARD* OR HARM* OR DEPEND*)
4. MH "SUBSTANCE USE DISORDERS" OR MH "HEROIN" OR MH "NARCOTICS" OR MH "DESIGNER DRUGS"
5. TX (DRUG OR SUBSTANCE OR POLYDRUG OR OPIOID OR OPIATE OR OPIUM OR HALLUCINOGEN OR COCAINE OR BENZODIAZEPINE* OR AMPHETAMINE* OR “ANTI-ANXIETY-AGENTS” OR BARBITURATE* OR “LYSERGIC ACID” OR KETAMINE OR CANNABIS OR MARIHUANA OR HASHISH OR INHALANT* OR SOLVENT OR STEROID* OR METHADONE OR MDMA OR ECSTASY OR MORPHINE) N3 (ABUSE* OR MISUSE* OR USE* OR PROBLEM* OR DEPEND* OR ADDICT* OR DISORDER* OR INTER* OR EDUCATION* OR PROGRAM*)

## 17. 12 OR 13 OR 14 OR 15 OR 16

1. MH "POSTOPERATIVE COMPLICATIONS+" OR MH "SURGERY, OPERATIVE" OR M "PREOPERATIVE

EDUCATION" OR MH "PREOPERATIVE PERIOD"OR MH "PREOPERATIVE CARE"

1. TX SURGICAL N3 TX (SETTING* OR PATIENT* OR OUTCOME*)
2. PREOP* N3 (INTERVENTION* OR EDUCATION OR PROGRAM* OR STUD* OR TREATMENT* OR PREVENT* OR THER* OR SERV* OR COUNSEL* OR SUPP* OR INFO* OR CONSULT* OR CARE)
3. PRE-OP* N3 (INTERVENTION* OR EDUCATION OR PROGRAM* OR STUD* OR TREATMENT* OR PREVENT* OR THER* OR SERV* OR COUNSEL* OR SUPP* OR INFO* OR CONSULT* OR CARE)
4. PERIOP* N3 (INTERVENTION* OR EDUCATION OR PROGRAM* OR STUD* OR TREATMENT* OR PREVENT* OR THER* OR SERV* OR COUNSEL* OR SUPP* OR INFO* OR CONSULT* OR CARE)
5. PERI-OP* N3 (INTERVENTION* OR EDUCATION OR PROGRAM* OR STUD* OR TREATMENT* OR PREVENT* OR THER* OR SERV* OR COUNSEL* OR SUPP* OR INFO* OR CONSULT* OR CARE)

## 24. 18 OR 19 OR 20 OR 21 OR 22 OR 23

## 25. 11 AND 17 AND 24

**COCHRANE**

1. MeSH descriptor Alcohol-Related Disorders explode all trees
2. MeSH descriptor Drinking Behavior explode all trees
3. (alcoholic* or alcoholism):ti,ab
4. ("alcohol* abuse" or "alcohol* misuse*" or "alcohol* use" or "alcohol* problem*" or "alcohol* depend*" or "alcohol* addict*" or "alcohol* disorder*"):ti,ab
5. MeSH descriptor Substance-Related Disorders, this term only
6. MeSH descriptor Amphetamine-Related Disorders, this term only
7. MeSH descriptor Cocaine-Related Disorders, this term only
8. MeSH descriptor Marijuana Abuse explode all trees
9. MeSH descriptor Opioid-Related Disorders explode all trees
10. MeSH descriptor Phencyclidine Abuse, this term only
11. MeSH descriptor Substance Abuse, Intravenous, this term only
12. MeSH descriptor Drug Users, this term only
13. ((narcotic* or heroin or opiate* or opioid* or opium or cocaine* or cannabis* or marijuana or marihuana or hashish or phencyclidine or benzodiaz* or barbiturate* or amphetamine* or MDMA or hallucinogen* or ketamine or lsd or inhalant* or substance*) near/1 (abuse* or misuse* or use* or problem* or depend* or addict* or disorder*)):ti,ab
14. ("drug abuse*" or "drug misuse*" or "drug problem*" or "drug depend*" or "drug addict*" or "drug disorder*" or "illicit drug*"):ti,ab

## 15. (#1 OR #2 OR #3 OR #4 OR #5 OR #6 OR #7 OR #8 OR #9 OR #10 OR #11 OR #12 OR #13 OR #14)

1. MeSH descriptor Postoperative Complications explode all trees
2. MeSH descriptor Surgical Procedures, Operative explode all trees
3. (surgical near (setting* or patient* or outcome*))
4. (PREOP* near (INTERVENTION* OR EDUCATION OR PROGRAM* OR STUD* OR TREATMENT* OR PREVENT* OR THER* OR SERV* OR COUNSEL* OR SUPP* OR INFO* OR CONSULT* OR CARE))
5. (PRE-OP* near (INTERVENTION* OR EDUCATION OR PROGRAM* OR STUD* OR TREATMENT* OR PREVENT* OR THER* OR SERV* OR COUNSEL* OR SUPP* OR INFO* OR CONSULT* OR

CARE))

1. (PERIOP* near (INTERVENTION* OR EDUCATION OR PROGRAM* OR STUD* OR TREATMENT* OR PREVENT* OR THER* OR SERV* OR COUNSEL* OR SUPP* OR INFO* OR CONSULT* OR CARE))
2. (PERI-OP* near (INTERVENTION* OR EDUCATION OR PROGRAM* OR STUD* OR TREATMENT* OR PREVENT* OR THER* OR SERV* OR COUNSEL* OR SUPP* OR INFO* OR CONSULT* OR CARE))

## 23. (#16 OR #17 OR #18 OR #19 OR #20 OR #21 OR #22)

## 24. (#15 AND #23)
